# Misprocessing of *α*-Galactosidase A, Endoplasmic Reticulum Stress, and the Unfolded Protein Response

**DOI:** 10.1681/ASN.0000000535

**Published:** 2024-11-12

**Authors:** Martina Živná, Gabriela Dostálová, Veronika Barešová, Dita Mušálková, Klára Svojšová, Doria Meiseles, Sara Kinstlinger, Ladislav Kuchař, Befekadu Asfaw, Helena Poupětová, Hana Vlášková, Tereza Kmochová, Petr Vyleťal, Hana Hartmannová, Kateřina Hodaňová, Viktor Stránecký, Lenka Steiner-Mrázová, Aleš Hnízda, Jan Živný, Martin Radina, Miroslav Votruba, Jana Sovová, Helena Trešlová, Larisa Stolnaja, Petra Reková, Lenka Roblová, Eva Honsová, Ivan Rychlík, Moran Dvela-Levitt, Anthony J. Bleyer, Aleš Linhart, Jakub Sikora, Stanislav Kmoch

**Affiliations:** 1 Research Unit for Rare Diseases, Department of Pediatrics and Inherited Metabolic Disorders, First Faculty of Medicine, Charles University in Prague and General University Hospital, Prague, Czech Republic; 2 Section on Nephrology, Wake Forest School of Medicine, Winston-Salem, North Carolina; 3 Second Department of Internal Cardiovascular Medicine, First Faculty of Medicine, Charles University and General University Hospital, Prague, Czech Republic; 4 The Mina and Everard Goodman Faculty of Life Sciences, Bar-Ilan University, Ramat-Gan, Israel; 5 Department of Neurology and Centre of Clinical Neuroscience, First Faculty of Medicine, Charles University and General University Hospital in Prague, Czech Republic; 6 Diagnostic Laboratory, Department of Pediatrics and Inherited Metabolic Disorders, General University Hospital, Prague, Czech Republic; 7 AeskuLab Pathology, Prague, Czech Republic; 8 Institute of Pathology, First Faculty of Medicine, Charles University and General University Hospital, Prague, Czech Republic; 9 Department of Medicine, Third Faculty of Medicine, Charles University in Prague and Faculty Hospital Kralovske Vinohrady, Prague, Czech Republic

**Keywords:** Fabry disease, pathogenesis, endoplasmic reticulum stress, unfolded protein response

## Abstract

**Key Points:**

- The clinical significance of a number of missense variants of *α*-galactosidase A is often ambiguous.
- Defective proteostasis of some missense *α*-galactosidase A variants induced chronic endoplasmic reticulum stress and the unfolded protein response.
- Endoplasmic reticulum stress and the unfolded protein response may explain clinical manifestations of non-classic Fabry disease.

**Background:**

Classic Fabry disease is caused by *GLA* mutations that result in loss of enzymatic activity of *α*-galactosidase A, lysosomal storage of globotriaosylceramide, and a resulting multisystemic disease. In non-classic Fabry disease, patients have some preserved *α*-galactosidase A activity and a milder disease course. Heterozygous female patients may also be affected. While Fabry disease pathogenesis has been mostly attributed to catalytic deficiency of mutated *α*-galactosidase A, lysosomal storage, and impairment of lysosomal functions, other pathogenic factors may contribute, especially in nonclassic Fabry disease.

**Methods:**

We characterized the genetic, clinical, biochemical, molecular, cellular, and organ pathology correlates of the p.L394P *α*-galactosidase A variant that was identified initially in six individuals with kidney failure by the Czech national screening program for Fabry disease and by further screening in an additional 24 family members.

**Results:**

Clinical findings in affected male patients revealed a milder clinical course, with approximately 15% residual *α*-galactosidase A activity with normal plasma lyso-globotriaosylceramide levels and abnormally low ratio of these values. None of the four available kidney biopsies showed lysosomal storage. Laboratory investigations documented intracellular retention of mutated *α*-galactosidase A with resulting endoplasmic reticulum stress and the unfolded protein response, which were alleviated with BRD4780, a small molecule clearing misfolded proteins from the early secretory compartment. We observed similar findings of endoplasmic reticulum stress and unfolded protein response in five kidney biopsies with several other classic and non-classic Fabry disease missense *α*-galactosidase A variants.

**Conclusions:**

We identified defective proteostasis of mutated *α*-galactosidase A resulting in chronic endoplasmic reticulum stress and unfolded protein response of *α*-galactosidase A expressing cells as a contributor to Fabry disease pathogenesis.

## Visual Abstract

**Figure:**
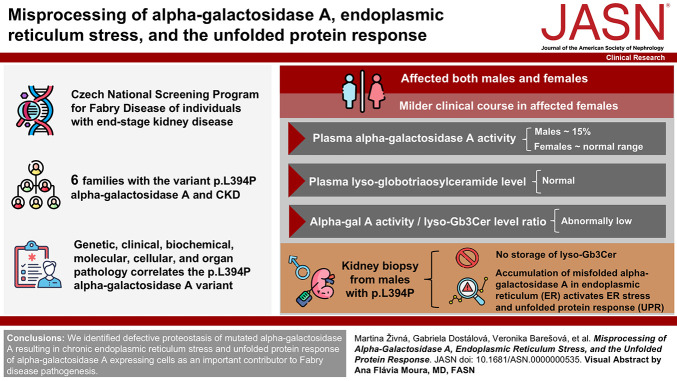

## Introduction

Fabry disease (OMIM #301500) is an X-linked condition caused by pathogenic variants of the *GLA* gene that result in the absence or enzymatic deficiency of *α*-galactosidase A. This enzyme defect leads to lysosomal storage of globotriaosylceramide in a variety of cell types and manifests as a multisystemic disease affecting the heart, kidney, blood vessels, peripheral nervous system, skin, and eyes.

Classic Fabry disease is best exemplified by male patients with loss-of-function *GLA* variants leading to loss of the *α*-galactosidase A enzyme activity, increased plasma lyso-globotriaosylceramide levels, and characteristic lysosomal storage vacuoles in affected cell types/tissues. Affected individuals typically develop cardiac, kidney, and neurologic complications in the third or fourth decade of life.^1^ Many, but not all, patients respond to enzyme replacement therapy with recombinant *α*-galactosidase A,^2^ although the response is suboptimal.^3^ Migalastat (deoxygalactonojirimycin), a small-molecule chaperone that facilitates *α*-galactosidase A trafficking from the endoplasmic reticulum to lysosomes for certain (amenable) mutated enzymes, is also helpful in some cases.^4,5^

Over the last two decades, an increasing number of patients have been described with non-classic Fabry disease.^6^ Many of these individuals have been identified by genetic screening of newborns or patient populations,^7^ allowing the identification of new *GLA* variants and corresponding clinical manifestations.^8,9^ These individuals typically have missense *GLA* variants, varying levels of *α*-galactosidase A activity, and a modest increase in plasma lyso-globotriaosylceramide. The phenotype occurs later in life and is often limited to cardiac or kidney disease.^10^ Lysosomal storage is less pronounced than in classic Fabry disease.^11^

Manifestations of Fabry disease have also been reported in female patients heterozygous for pathogenic *GLA* variants. Clinical symptoms occur at a later age than in male patients and often do not correlate with *α*-galactosidase A activity,^12^ with higher rates of symptomatic disease than expected by random X-inactivation.^13^

While the pathogenesis of Fabry disease has been mostly attributed to *α*-galactosidase A deficiency, lysosomal globotriaosylceramide accumulation, and impairment of lysosomal functions,^14^ other factors may contribute.^15–17^

*α*-galactosidase A is synthesized and delivered to the lysosome through the secretory pathway.^18^ Specific *GLA* variants might affect the synthesis, processing, and stability of *α*-galactosidase A in different ways.^19^ While misfolding of some *α*-galactosidase A variants and their degradation through endoplasmic reticulum–associated protein degradation (ERAD) constitute the established mechanism leading to lysosomal enzyme deficiency,^20^ the potential contribution of *α*-galactosidase A misprocessing, intracellular retention and consequent endoplasmic reticulum stress, and unfolded protein response to development of Fabry disease symptoms have been investigated only *in vitro*.^21,22^

To this end, in this work, we characterized the clinical and pathophysiological correlates of the *GLA* variant c.1181T>C encoding a missense variant p.L394P of *α*-galactosidase A that was found in a nationwide Fabry disease screening program of Czech dialysis patients.

## Methods

### Patients

The probands were identified through a Fabry disease screening program of 6352 patients (94% of the dialysis population) in the Czech Republic. *α*-galactosidase A activity,^23^ lyso-globotriaosylceramide,^24^ and genetic investigations were performed as described earlier.^25^

### Degradation of Globotriaosylceramide in Cultured Skin Fibroblasts

Skin fibroblasts were loaded with mass-labeled C23:0,d18:1 globotriaosylceramide. Conduritol B epoxide (Calbiochem-Novabiochem GmbH, Germany) was added to block metabolic conversion of glucosylceramide to ceramide. Sphingolipids were extracted from the cell homogenate and quantified using the mass spectrometry as previously described.^26,27^

### Histopathological, Immunohistochemical, and Ultrastructural Electron Microscopic Analyses of Kidney Biopsies

*α*-galactosidase A, CRELD2, HSPA5/BiP/GRP78, PDIA4/ERp72, and lysosomes were immunodetected with antigen-specific antibodies and peroxidase detection (Supplemental Table 1). Ultrastructural studies were performed as reported earlier.^28^

### Intracellular Localization of *α*-Galactosidase A in Kidney Biopsies, Skin Fibroblasts, and Transfected HEK 293 Cells

*α*-galactosidase A and cellular compartments were detected with antigen-specific antibodies and corresponding fluorescently labeled antibodies (Supplemental Table 1). Confocal microscopy, fluorescence image acquisition, and analysis have been performed as previously described.^29^ Relative subcellular distribution was calculated from the mean of *α*-galactosidase A signal intensities retrieved from pixels with positive colocalization signals. The signals were obtained on average from about 50 cells with 5000–15,000 events identified.

### *α*-Galactosidase A Expression in Transfected HEK 293 Cells

Wild-type *GLA* was cloned into pCR3.1 and corresponding mutated constructs were prepared by site-directed mutagenesis. Transfection were performed using Lipofectamine 3000 (Invitrogen, Paisley, United Kingdom). *α*-galactosidase A-FLAG was detected by Western blot analysis, and its intracellular localization was assessed by immunofluorescence and confocal microscopy as described above.

### RNA Sequencing of HEK 293 Cells Stably Expressing *α*-Galactosidase A-FLAG

Stranded mRNA-Seq libraries were prepared with the KAPA mRNA HyperPrep Kit for Illumina Platforms (Roche). Paired-end reads of 2×100 base pairs were sequenced on Illumina NovaSeq6000. The resulting FASTQ files were trimmed using Atropos.^30^ Gene-level abundances were estimated using Salmon^31^ Normalization, and differential expression analyses were performed within DESeq2 R package.^32^

### Proteomic Analysis of HEK 293 Cells Stably Expressing *α*-Galactosidase A-FLAG

Trypsin-generated peptides were analyzed with tandem mass spectrometry, quantified with the MaxQuant software^33^ and analyzed using Perseus 1.6.1.3 software.^34^ Pathway analysis of the proteomic and RNASeq data were performed with GSEA.^35^

### qRT-PCR Analysis of Unfolded Protein Response Markers in Transfected HEK 293 Cells

RNA was reverse transcribed and amplified using the SOLIScript one-step Multiplex Probe Kit (Solis Biodyne). Relative expression levels were calculated by 2^−∆∆Ct^ method using *PUM1*, *CASC3*, *UBC*, and *TBP* genes as a reference. Primer and probe sequences used are listed in Supplemental Table 2.

### BRD4780 and Migalastat Treatment of HEK 293 Cells Stably Expressing *α*-Galactosidase A Variants

HEK 293 cells were cultured initially in standard media for 24 hours and then in media containing 10 *µ*M BRD4780 (AGN 192403 hydrochloride, CAS number: 1021868-90-5, Tocris), 50 *µ*M deoxygalactonojirimycin (Deoxygalactonojirimycin hydrochloride, Migalastat, CAS number 75172-81-5, Merck), or 10 *µ*l of DMSO for 48 hours. The qRT-PCR analysis of unfolded protein response markers was performed in cell lysates as described above. Intracellular content of *α*-galactosidase A-FLAG, BiP, GRP94, and ERp72 was assessed by Western blot. Intracellular localization of *α*-galactosidase A-FLAG was assessed by immunofluorescence and confocal microscopy as described above. *α*-galactosidase A activities in cell lysate and culture media were measured as described above.

Detailed protocols for all methods are provided in the Supplemental Methods.

## Results

### Patients

**Figure 1:**
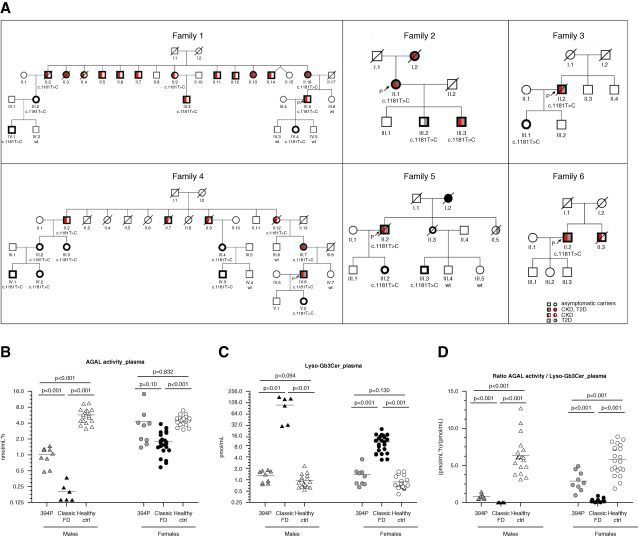
**Family pedigrees with biochemical characteristics.** (A) Generations and individuals are identified successively by Roman and Arabic numerals, respectively. Black bolded symbols denote genetically affected individuals with the c. 1181T>C variant. Red and gray coloring denote presence of CKD and type 2 diabetes mellitus. White coloring denotes asymptomatic carriers. Open symbols denote clinically unaffected individuals; wt denotes absence of the *GLA* mutation; if not stated, DNA was unavailable for investigation. (B) *α*-galactosidase A activities, (C) lyso-globotriaosylceramide levels, and (D) corresponding values of their ratio in plasma of male patients and female patients with the 394P *α*-galactosidase A (*n*=9 and *n*=9, respectively), classic Fabry disease variants (*n*=6 and *n*=23, respectively), and healthy controls (*n*=19 and *n*=21, respectively). Data are means±SEM. The statistical significance of the differences between the categories is represented by *P* values calculated by two-tailed *t* test. ctrl, control; FD, Fabry disease.

As part of the Czech nationwide Fabry disease screening program of dialysis patients, six probands were identified with approximately 15% residual blood *α*-galactosidase A activity and the c.1181T>C *GLA* variant, encoding missense p.L394P *α*-galactosidase A. Family histories revealed numerous individuals with CKD and type 2 diabetes mellitus (Figure 1A). Whole genome sequencing performed in F1_III.5 and F3_III.1 revealed 4.7 Mb of shared genetic material on chromosome X (100016210–104743750; [hg19]), confirmed the expected founder effect, and excluded other potential disease-causing variants. This variant has been identified in two individuals of European (non-Finnish) population in the Genome Aggregation Database. It is not conserved in vertebrate species and is classified as a variant of uncertain significance according to American College of Medical Genetics recommendations.^36^

Family screening identified 30 carriers (15 male carriers and 15 female carriers) with the c.1181T>C variant. There was a high prevalence of Fabry disease symptoms in this group (Supplemental Table 3). Laboratory investigations revealed that all of nine investigated male carriers had low *α*-galactosidase A activities in plasma (Figure 1B) and leukocytes (Supplemental Figure 3), with normal plasma lyso-globotriaosylceramide levels (Figure 1C) but abnormally low ratio (Figure 1D). Nine female carriers had normal plasma and leukocyte *α*-galactosidase A activities, with two of them having low plasma *α*-galactosidase A activities. All female carriers had normal plasma lyso-globotriaosylceramide levels. The ratio of plasma enzyme activity to lyso-globotriaosylceramide levels was abnormal in some individuals (Figure 1D).

Twenty-three (75%) carriers had at least one prespecified Fabry disease manifestation. The most frequent findings included the presence of proteinuria (*n*=17), CKD (*n*=10, including seven patients with kidney failure), gastrointestinal symptoms (*n*=10), ocular changes (*n*=7), left ventricular hypertrophy (*n*=7), clinical manifestations attributable to small fiber neuropathy (*n*=6), and stroke/transient ischemic attack history (*n*=3). Only one carrier (a female) had typical Fabry-related angiokeratomas. Hypertension was present in nine individuals. Type 2 diabetes mellitus was present in 12 genetically affected individuals and no genetically unaffected family members.

**Figure 2:**
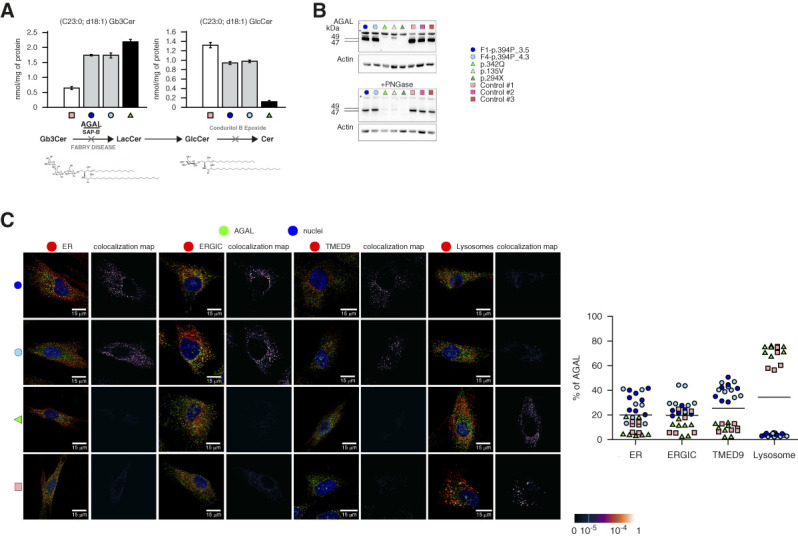
**Degradation of globotriaosylceramide and *α*-galactosidase A expression in cultured skin fibroblasts.** (A) Skin fibroblasts were loaded with mass-labeled C23:0,d18:1 globotriaosylceramide. Conduritol B epoxide, a covalent inhibitor of GlcCer-*β*-glucosidase, was applied to block the downstream metabolic conversion of glucosylceramide to ceramide. After 4 days of incubation, the original substrate, C23:0,d18:1 globotriaosylceramide, and the resulting product, C23:0,d18:1 glucosylceramide, were isolated from cell homogenates and quantified by FIA-ESI-MS/MS. Compared with control cells, degradation of the C23Gb3Cer was reduced in cells from two males with the 394P *α*-galactosidase A, but to a lesser extent than in cells of the male with classic Fabry disease and 342Q *α*-galactosidase A. (B) Immunoblot analysis of *α*-galactosidase A in cell lysates demonstrating that the synthesis and post-translational processing of the 394P *α*-galactosidase A is similar to male controls. In skin fibroblasts from male patients with classic Fabry disease, the amount of *α*-galactosidase A is lower. (C) To assess whether and how identified *α*-galactosidase A mutations affect intracellular localization, we detected *α*-galactosidase A (green) and colocalized the resulting immunofluorescent signal with markers for lysosomes (LAMP2; red) and endoplasmic reticulum (PDI; red), endoplasmic reticulum–Golgi intermediate compartment (ERGIC53; red) and the secretory cargo receptor TMED9 (red) in cultured skin fibroblasts. Nuclei are in blue. In affected skin fibroblasts from individuals with the 394P mutation (F1-394P_3.5 and F4-394P_4.3, see pedigrees), *α*-galactosidase A localizes mainly to the endoplasmic reticulum and endoplasmic reticulum–Golgi intermediate compartment and colocalizes with TMED9 with minimal staining of the lysosome. In skin fibroblasts from a classic Fabry disease patient with the 342Q mutation and from an unaffected control, *α*-galactosidase A localized mostly to lysosomes with minimal staining in other compartments. The degree of *α*-galactosidase A colocalization with selected markers is demonstrated by the fluorescent signal overlap coefficient values that range from 0 to 1. The resulting overlap coefficient values are presented as the pseudo color whose scale is shown in the corresponding lookup tables. Subcellular distribution of *α*-galactosidase A in selected compartments is shown in the adjacent graph. The values represent the percentage of the total of mean *α*-galactosidase A colocalization signal intensities in corresponding compartments. On average, 50 cells were analyzed and 5000–15,000 events were identified for each sample. ER, endoplasmic reticulum; ERGIC, endoplasmic reticulum–Golgi intermediate compartment; LacCer, lactosylceramide; SAP-B, saposin B; TMED9, transmembrane P24 trafficking protein 9.

Loading studies revealed that the degradation of C23:0,d18:1 globotriaosylceramide was lower in cultured skin fibroblasts from two male patients with the 394P *α*-galactosidase A, but to a lesser extent than in fibroblasts of the male with classic Fabry disease (Figure 2A). The synthesis and processing of the 394P *α*-galactosidase A seemed unaffected (Figure 2B). However, immunofluorescence studies revealed that 394P *α*-galactosidase A was retained in compartments of the early secretory pathway with minimal but noticeable staining in lysosomes. In control fibroblasts and fibroblasts of a male with classic Fabry disease, *α*-galactosidase A localized largely to lysosomes (Figure 2C).

**Figure 3:**
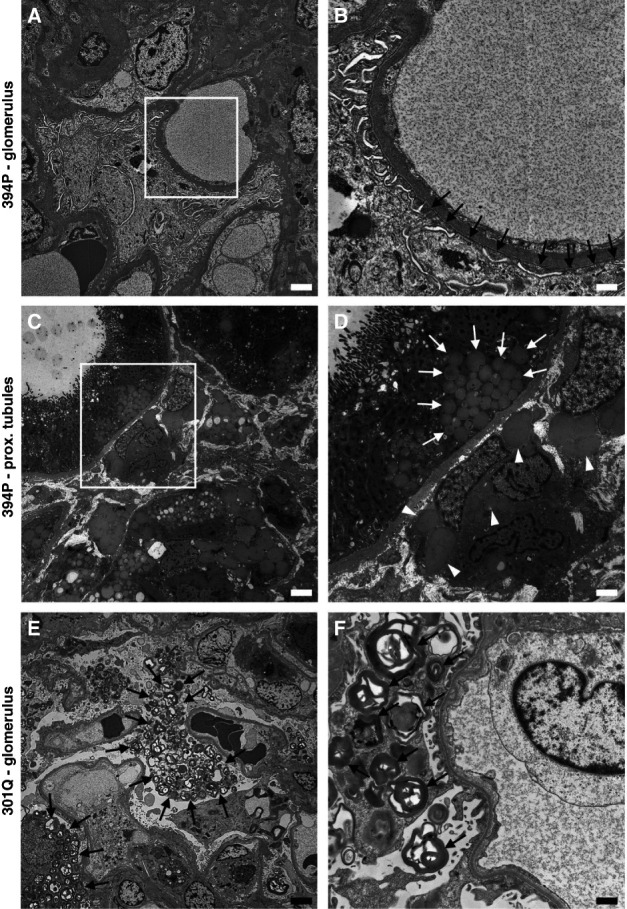
**Kidney ultrastructural pathology.** (A and B) Glomerular structures in a male with the 394P *α*-galactosidase A variant. Podocytes and endothelial cells do not contain typical Fabry disease storage lysosomes. Partial pedicel effacement is shown in higher detail in (B, black arrows). The white square in (A) corresponds to the area shown in (B). (C and D) Non–membrane-bound lipid droplets (white arrows) were present in proximal tubular epithelial and interstitial cells (white arrowheads) of a male with the 394P *α*-galactosidase A as a likely secondary structural abnormality linked to (lipo)proteinuria. Notably, lysosomal storage typical for Fabry disease was not found in any cell type present in the tested tissue. The white square in (C) corresponds to the area shown in (D). (E and F) Characteristic multilamellar storage lysosomes (black arrows) are abundant in podocytes of a male with classic Fabry disease and the 301Q *α*-galactosidase A. The white square in (E) corresponds to the area shown in (F). Scale bars: (A, D, and E) 2 *μ*m; (C) 5 *μ*m; (B and F) 750 nm. prox. tubules, proximal tubules.

**Figure 4:**
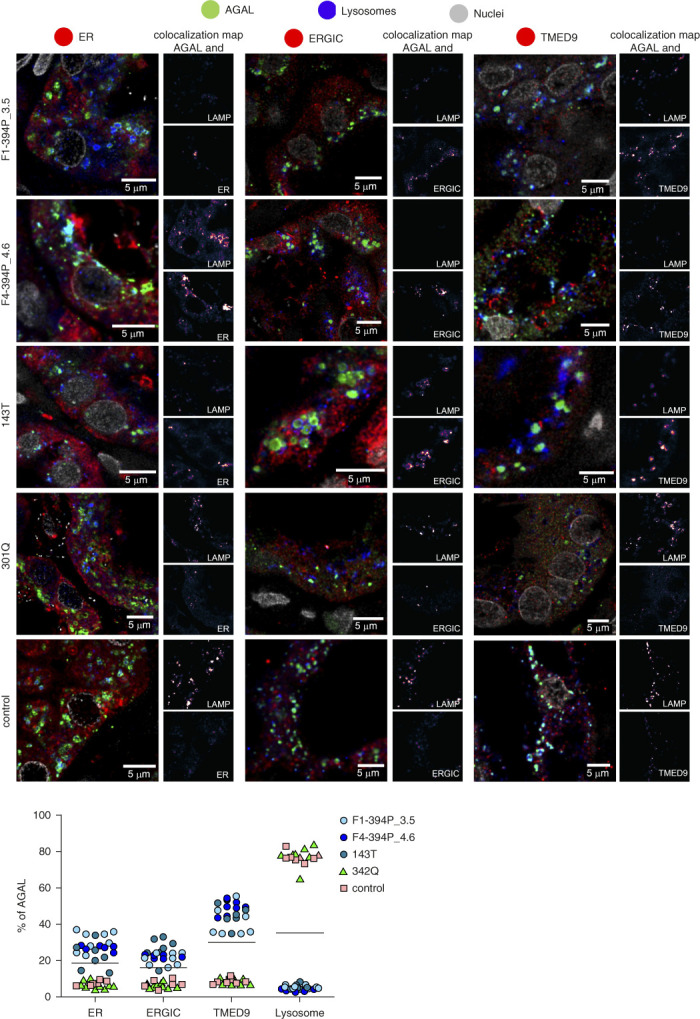
***α*-galactosidase A localization in kidney.** To assess whether and how the identified variants affect *α*-galactosidase A intracellular localization in tubular epithelial cells, we detected *α*-galactosidase A (green) and colocalized the resulting immunofluorescent signal with markers of lysosomes (LAMP2; blue) and endoplasmic reticulum (PDI; red), endoplasmic reticulum–Golgi intermediate compartment (ERGIC53; red), and the secretory cargo receptor TMED9 (red) in kidney biopsies. Nuclei are in gray. In kidney tissue from the male with the 394P variant (F1-394P_III.5 and F4-394P_IV.6, see pedigrees), *α*-galactosidase A localizes mainly to endoplasmic reticulum and endoplasmic reticulum–Golgi intermediate compartment and colocalizes with TMED9 with minimal staining of lysosomes. A similar pattern of *α*-galactosidase A distribution was found in the kidney from a female with a 143T variant. In a kidney biopsy of a male with classic Fabry disease and 301Q pathogenic variant and in a control kidney, *α*-galactosidase A localized mostly to lysosomes with minimal staining in other compartments. The degree of *α*-galactosidase A colocalization with selected markers is demonstrated by the fluorescent signal overlap coefficient values that range from 0 to 1. The resulting overlap coefficient values are presented as the pseudo color whose scale is shown in the corresponding lookup tables. Subcellular distribution of *α*-galactosidase A in selected compartments are shown in the graph below. The values represent the percentage of the total of mean *α*-galactosidase A colocalization signal intensities in corresponding compartments. On average, 50 cells were analyzed and 5000–15,000 events were identified for each sample.

Histopathologic and immunohistochemical studies in kidney biopsies of two male patients with the 394P *α*-galactosidase A revealed enlarged glomeruli without proliferation and mild focal interstitial fibrosis. Characteristic lysosomal storage found in classic Fabry disease (Supplemental Figure 1) was not detected in glomeruli. Ultrastructural electron microscopic studies in kidney biopsies of male patients with the 394P variant showed partial effacement of podocyte pedicels and non–membrane-bound lipid droplets in proximal tubular epithelium and interstitial cells. Lysosomal storage typical for Fabry disease was not found in any cell type present in the tested tissue (Figure 3). Immunofluorescence studies in kidney biopsies of patients and controls with classic Fabry disease showed the usual distribution of *α*-galactosidase A with high expression in tubular epithelial cells and minimal podocyte expression.^37^ The mutated *α*-galactosidase A localized mainly to the endoplasmic reticulum and endoplasmic reticulum–Golgi intermediate compartment and colocalized with transmembrane P24 trafficking protein 9 (TMED9), whereas in a control kidney and a kidney biopsy of a male with classic Fabry disease, *α*-galactosidase A localized to lysosomes (Figure 4).

Given the clinical presentation, histopathologic, ultrastructural, and fibroblast culture findings, we reasoned that misprocessing with abnormal intracellular distribution of the 394P *α*-galactosidase A might disrupt the secretory pathway, activate the endoplasmic reticulum stress with unfolded protein response, and cause kidney disease, similar to several other protein-misfolding disorders.^38–41^ At the same time, a portion of the mutated *α*-galactosidase A might escape degradation by secretory pathway quality control mechanisms and transit to lysosomes, where its residual enzymatic activity might prevent intracellular accumulation of globotriaosylceramide and related glycosphingolipids that is characteristic in Fabry disease.

**Figure 5:**
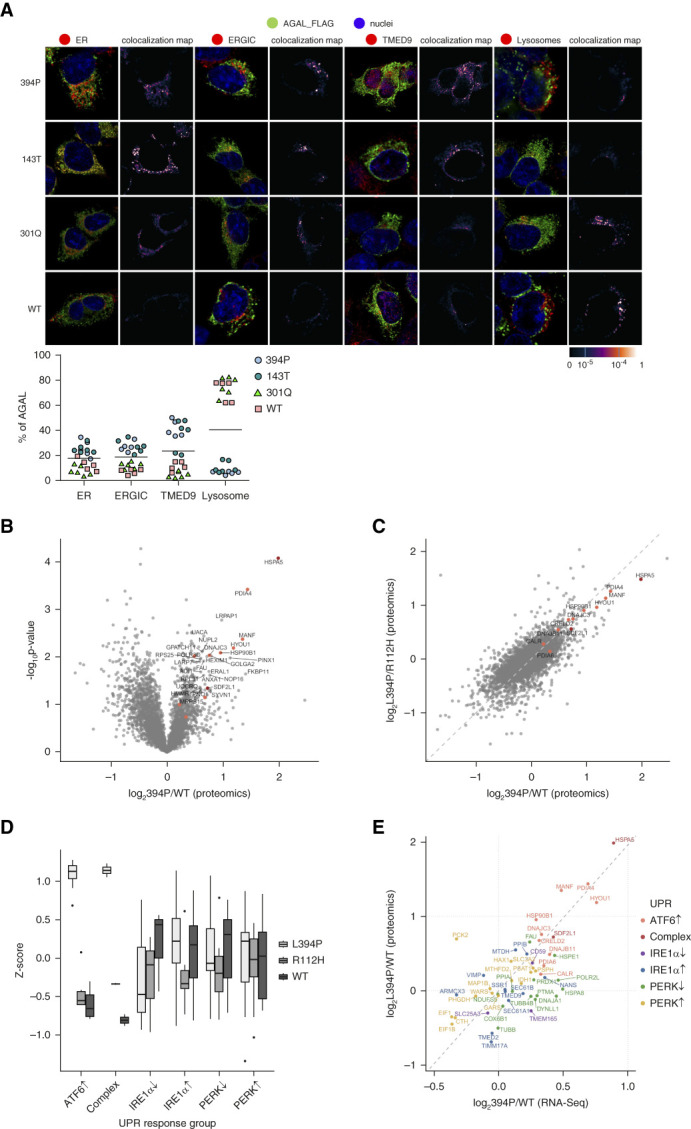
**Pathogenic consequences of the 394P *α*-galactosidase A.** (A) Consistent with kidney biopsies, the corresponding *α*-galactosidase A-FLAG proteins stably expressed in HEK 293 also had a different intracellular distribution. We detected *α*-galactosidase A-FLAG (green) and colocalized the resulting immunofluorescent signal with markers of endoplasmic reticulum (PDI; red), endoplasmic reticulum–Golgi intermediate compartment (ERGIC53; red), the secretory cargo receptor (TMED9; red), and lysosomes (LAMP2; red). Nuclei are stained in blue. The 394P and 143T *α*-galactosidase A-FLAG proteins localized mainly to the endoplasmic reticulum and endoplasmic reticulum–Golgi intermediate compartment and colocalized with TMED9 with minimal staining of the lysosomes. The 301Q (classic Fabry mutation) and the wild-type *α*-galactosidase A-FLAG proteins localized mostly to lysosomes with minimal staining in other compartments. The degree of *α*-galactosidase A colocalization with selected markers is demonstrated by the fluorescent signal overlap coefficient values ranging from 0 to 1. Subcellular distribution of *α*-galactosidase A in selected compartments is shown in the graph below. The values represent the percentage of the total mean *α*-galactosidase A colocalization signal intensities in corresponding compartments. On average, 50 cells were analyzed and 5000–15,000 events were identified for each sample. (B) Protein composition of HEK 293 cells stably expressing 394P, 112H (classic Fabry), and wild-type *α*-galactosidase A-FLAG proteins was analyzed using mass spectrometry. The binary logarithm of the ratios of individual protein amounts between the cells expressing 394P-FLAG and wild-type–FLAG proteins (log2[394P/wild-type]) and the decadic logarithm of the nominal probability that the protein is differentially expressed (−log10 [raw *P* value]) are visualized using a volcano plot. Proteins on the right (positive) side of the *x* axis are increased in the cells expressing the 394P *α*-galactosidase A-FLAG. Red dots represent proteins involved in the unfolded protein response. (C) Correlation of individual protein amount ratios in HEK 293 cells stably expressing 394P with 112H (classic Fabry) (log2[394P/112H]) and wild-type *α*-galactosidase A-FLAG proteins (log2[394P/wild-type]) shows that the presence of the 394P-FLAG specifically increases the amount of protein involved in the unfolded protein response (red dots). (D) Specific activation of activating transcription factor 6 and complex branches of the unfolded protein response in HEK 293 cells expressing the 394P *α*-galactosidase A-FLAG. Z-scores of normalized protein expression values were obtained from proteomic analysis of HEK 293 cells stably expressing 394P, 112H (classic Fabry) and wild-type *α*-galactosidase A-FLAG proteins. (E) Correlation of protein amounts and mRNA levels of individual unfolded protein response components in HEK 293 cells expressing the 394P and wild-type *α*-galactosidase A-FLAG. UPR, unfolded protein response; WT, wild-type.

Accordingly, *in silico* analysis revealed that the L394 residue is located outside of the *α*-galactosidase A active site, indicating that enzymatic activity may be structurally maintained despite potential issues with protein misfolding (Supplemental Figure 2). Consistent with immunofluorescent studies in skin fibroblasts and human kidney, the 394P *α*-galactosidase A with a C-terminal FLAG tag stably expressed in HEK 293 cells localized mainly to the endoplasmic reticulum and endoplasmic reticulum–Golgi intermediate compartment, with preferential codistribution with TMED9, and with a minor presence in lysosomes. Wild-type *α*-galactosidase A with a C-terminal FLAG tag and the classic Fabry disease–causing variant 301Q *α*-galactosidase A with a C-terminal FLAG tag localized mainly to lysosomes (Figure 5A). A small proportion of all the *α*-galactosidase A variants localized to the Golgi apparatus (data not shown). RNA sequencing (Figure 5B) and proteomic analyses (Figure 5C) of HEK 293 cells stably expressing the 394P *α*-galactosidase A-FLAG revealed activation of endoplasmic reticulum stress and unfolded protein response (Supplemental Tables 4–6), with specific upregulation of the activating transcription factor 6 and general (Complex) branches (Figure 5, D and E). This response was absent in HEK 293 cells stably expressing the wild-type or classic Fabry disease–causing variant 112H *α*-galactosidase A-FLAG. The proteins involved in the ribonuclease inositol-requiring protein-1 and the endoplasmic reticulum resident transmembrane protein kinase pathways showed minimal changes (Figure 5D). The initial step in inositol-requiring protein-1 response, the X-box binding protein 1 mRNA splicing, was also not activated (not shown). Consistently, expression of the endoplasmic reticulum stress markers, PDIA4/ERp72, HSPA5/BiP/GRP78, and endoplasmic reticulum stress and activating transcription factor 6 inducible protein CRELD2,^42^ were the most highly induced in kidney tubular cells of two male patients with the 394P variant, but to a lesser extent or not induced in five kidney biopsies with other missense variants causing Fabry disease and healthy control (Supplemental Figures 3–5).

After identification of abnormal intracellular trafficking and unfolded protein response activation with partial slip-through of the 394P *α*-galactosidase A to lysosomes, we explored similar changes in other *α*-galactosidase A variants associated with classic and non-classic Fabry disease (Supplemental Table 7).

**Figure 6:**
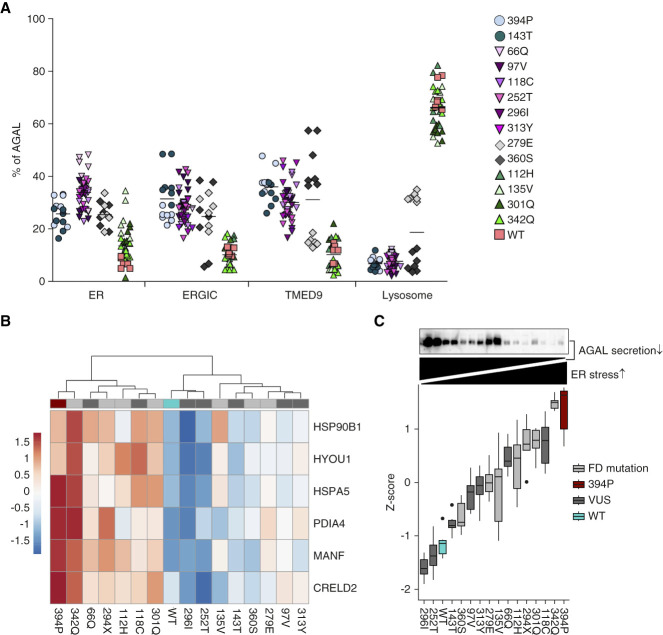
**Localization of *α*-galactosidase A-FLAG in transiently transfected HEK 293 cells.** (A) To assess how other nontruncating missense *α*-galactosidase A variants of unknown significance affect intracellular localization, we detected corresponding *α*-galactosidase A-FLAG proteins transiently expressed in HEK 293 cells and colocalized the resulting immunofluorescent signal with markers of endoplasmic reticulum (PDI), endoplasmic reticulum–Golgi intermediate compartment (ERGIC53), the secretory cargo receptor TMED9, and lysosomes (LAMP2). Subcellular distribution of *α*-galactosidase A in selected compartments is shown in the graph. The values represent the percentage of the total mean *α*-galactosidase A colocalization signal intensities in corresponding compartments. On average, 50 cells were analyzed and 5000–15,000 events were identified for each sample. (B) Unsupervised hierarchical clustering of normalized expression values (Z-scores) of selected endoplasmic reticulum stress and unfolded protein response markers obtained from RT-qPCR analyses of HEK 293 cells transiently expressing individual *α*-galactosidase A variants. The Z-scores are presented as the pseudocolor whose scale is shown in the corresponding lookup table. The 394P *α*-galactosidase A variant is shown as a dark red; *α*-galactosidase A variants associated with either classic or non-classic Fabry disease are shown in light gray; *α*-galactosidase A variants of unknown significance are shown in dark gray, wild-type *α*-galactosidase A is shown in blue. (C) Intensity of the endoplasmic reticulum stress shown as Z-scores of means of normalized mRNA expression values of selected endoplasmic reticulum stress and unfolded protein response markers calculated for each variant. The endoplasmic reticulum stress intensity (dark) is proportional to secretory incompetence of the corresponding variant shown above by immunodetection of *α*-galactosidase A in cell culture media.

In a kidney biopsy of a female with the p.A143T variant,^43^ *α*-galactosidase A had a similarly abnormal intracellular distribution to that of patients with the non-classic variant (Figure 4). Furthermore, renal tubular cells showed induction of CRELD2 expression but to a lesser extent than was detected in individuals with the 394P variant (Figure 6). Accordingly, in the HEK 293 cells stably expressing the 143T variant of *α*-galactosidase A with a C-terminal FLAG tag, the enzyme localized mainly to the endoplasmic reticulum–Golgi intermediate compartment with preferential codistribution with the cargo receptor TMED9, and with a minor presence in lysosomes (Figure 5).

Similar localization of *α*-galactosidase A to the endoplasmic reticulum–Golgi intermediate compartment and codistribution with TMED9 that were characteristic for 394P and 143T variants were observed also with several other *α*-galactosidase A variants either of unknown significance or identified in individuals with non-classic Fabry disease, when transiently expressed in HEK 293 cells (Supplemental Figure 6). *α*-galactosidase A variants associated with classic Fabry disease always localized to lysosomes (Figure 6A and Supplemental Figure 3). Irrespective of their intracellular distribution and phenotypic associations, most of the tested variants had limited *α*-galactosidase A secretion. The deficit in secretion was proportional to the intensity of the endoplasmic reticulum stress and unfolded protein response activation (Figure 6, B and C).

**Figure 7:**
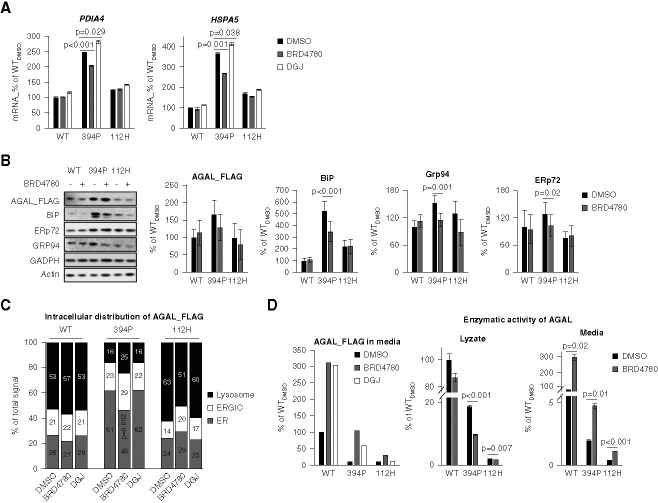
**Effects of BRD4780 and migalastat on *α*-galactosidase A-FLAG expression in stably transfected HEK 293 cells.** (A) In HEK 293 cells stably expressing the 394P *α*-galactosidase A-FLAG, BRD4780 treatment attenuated transcription of endoplasmic reticulum stress markers HSPA5/BiP/GRP78 and PDIA4/ERp72; means±SEM (*n*=3); *P* values were calculated with the two-tailed *t* test; migalastat (DGJ) had the opposite effect. (B) BRD4780 treatment had nonsignificant effect on intracellular content of the 394P *α*-galactosidase A-FLAG but attenuated expression of endoplasmic reticulum stress markers on protein level; means±SEM (*n*=5); *P* values were calculated by two-way ANOVA. (C) BRD4780 treatment facilitated trafficking of 394P *α*-galactosidase A-FLAG through the secretory pathway to lysosomes. The values represent the percentage of the total mean *α*-galactosidase A colocalization signal intensities in corresponding compartments. On average, 50 cells were analyzed and 5000–15,000 events were identified for each sample; means±SD; *P* values were calculated by two-tailed *t* test. (D) BRD4780 treatment resulted in increased secretion and enzymatic activity of 394P *α*-galactosidase A-FLAG, wild-type *α*-galactosidase A-FLAG, and 112H *α*-galactosidase A-FLAG into cell culture media. DGJ had positive effect on secretion of wild-type *α*-galactosidase A-FLAG and 394P *α*-galactosidase A-FLAG but not on that of 112H *α*-galactosidase A-FLAG; means±SEM (*n*=3 replicates); *P* values were calculated by two-tailed *t* test. BiP, binding immunoglobulin protein; DGJ, deoxygalactonojirimycin.

Recent research identified BRD4780 as a small compound that facilitates removal of unfolded proteins from TMED9-enriched transport vesicles into lysosomes and modulates the unfolded protein response.^39^ To this end, BRD4780 treatment of HEK 293 cells stably expressing 394P *α*-galactosidase A-FLAG attenuated transcription and expression of endoplasmic reticulum stress markers HSPA5/BiP/GRP78 and PDIA4/ERp72 (Figure 7, A and B) and facilitated trafficking of *α*-galactosidase A through the secretory pathway (Figure 7C), resulting in increased secretion and enzymatic activity in cultured media (Figure 7D). The treatment with BRD4780 had no effect on transcription and expression of endoplasmic reticulum stress markers in HEK 293 cells stably expressing the wild-type *α*-galactosidase A-FLAG or classic Fabry disease–causing variant 112H *α*-galactosidase A-FLAG. Nevertheless, the treatment facilitated trafficking and increased secretion of *α*-galactosidase A similarly to 394P *α*-galactosidase A-FLAG (Figure 7).

Treatment with migalastat (deoxygalactonojirimycin), a pharmacological chaperone that stabilizes and facilitates trafficking of amenable nontruncating *α*-galactosidase A variants from the endoplasmic reticulum to lysosomes, increased biosynthesis and secretion of the wild-type *α*-galactosidase A-FLAG and 394P *α*-galactosidase A-FLAG but not that of 112H *α*-galactosidase A-FLAG. Transcription of endoplasmic reticulum stress markers in HEK 293 cells stably expressing 394P *α*-galactosidase A-FLAG was even augmented (Figure 7A).

## Discussion

In this work, we characterized the clinical, biochemical, genetic, molecular, cellular, and organ pathology correlates of the 394P *α*-galactosidase A variant identified in six individuals through a Czech national screening program for Fabry disease in dialysis patients. Additional 24 carriers of this variant were identified by genetic screening of affected families. The genetically affected individuals presented predominantly with proteinuria, hypertension, CKD, and heart disease. The median age of kidney failure was older than that of classic Fabry disease. Characteristic findings such as angiokeratomas and neuropathy were less commonly seen. Hypertension was present in 40%, consistent with prior reports.^44^ Interestingly, type 2 diabetes mellitus was detected in 40% of individuals diagnosed with the 394P *α*-galactosidase A variant versus 0% of family members who did not have this variant. Other reports have noted a similar prevalence of type 2 diabetes mellitus in Fabry disease and the general population.^45^ The increased prevalence noted in this study could be coincidental or somehow related to the 394P variant and will require further investigation in non-classic Fabry disease. Kidney biopsies did not reveal evidence of diabetic nephropathy, and patients with the 394P variant who did not have type 2 diabetes mellitus also proceeded to CKD. It is likely that expression of some *α*-galactosidase A variants may induce endoplasmic reticulum stress and unfolded protein response also in *β*-cells. This potential mechanism has been identified in more than 40 genes with mutations reported to cause monogenic diabetes, 11 of which directly or indirectly induce endoplasmic reticulum stress in pancreatic *β*-cell.^46^ Women had milder phenotype with later age at onset than male patients. Both, affected male patients and female patients, presented with significantly lower plasma ratios of *α*-galactosidase A activity to lyso-globotriaosylceramide, which is suggestive of functional impairment of the enzyme with an effect on glycosphingolipid metabolism. Also in cultured male skin fibroblasts, the residual activity of 394P *α*-galactosidase A resulted in decreased globotriaosylceramide degradation that was intermediate between control and classic Fabry disease fibroblasts, likely explaining normal plasma lyso-globotriaosylceramide levels in affected carriers. Accordingly, characteristic lysosomal storage was absent in all kidney cell types.^47^ Biopsy specimen, however, showed podocytopathy (effacement of podocyte pedicels), non–membrane-bound droplets in proximal tubular epithelium and interstitial cells, and minimal interstitial fibrosis. Interestingly, pedicel effacement was very similar to globotriaosylceramide-independent podocyte changes reported in the zebrafish *α*-galactosidase A deficient model,^15^ thus providing further evidence for lysosomal storage–independent mechanism of Fabry disease pathogenesis.

The pathogenesis and clinical manifestations of classic Fabry disease have been primarily attributed to the absence or profound enzymatic deficiency of *α*-galactosidase A, accumulation of globotriaosylceramide, and consequent disarrangement(s) of intracellular pathways linked to lysosomal (dys)function(s).^14^ Individuals with classic Fabry disease commonly carry truncating *GLA* variants resulting in loss of *α*-galactosidase A and/or its enzymatic activity. However, approximately 50% of reported pathogenic *GLA* variants are missense variants.^10^ Many of these variants result in non-classic Fabry disease, with lesser organ involvement, later age of onset, and >5% of residual enzyme activity.^6^ Moreover, many *GLA* variants of likely pathogenicity or uncertain significance have been identified within screening programs for Fabry disease in newborns and high-risk populations.^7^ In many individuals affected with these variants and presenting with atypical clinical features of Fabry disease, other factors may be contributing to disease pathogenesis besides the *α*-galactosidase A enzymatic deficiency and lysosomal storage.

For many missense *α*-galactosidase A variants that are associated with the classic Fabry disease, enzyme deficiency results from misfolding and ERAD.^48^ In amenable variants, pharmacological chaperones allow correct folding, stabilization, and trafficking of mutated *α*-galactosidase A proteins through the secretory pathway to the lysosomes to catabolize accumulated substrates.^4,49,50^

Instead of being prematurely degraded or transported to the lysosome as in classic Fabry disease, most of the 394P *α*-galactosidase A localizes to endoplasmic reticulum–Golgi intermediate compartment and TMED9 positive cargo receptor-containing vesicles, where the protein folding quality control system proceeds. The interference of misprocessed *α*-galactosidase A with the proper function of the secretory pathway leads to the endoplasmic reticulum stress and activation of the unfolded protein response, presumably triggering effects with pathogenic consequences on secretory active and/or postmitotic cell types. Similar dearrangements have been identified as primary in various monogenic glomerular and tubular diseases,^41^ endothelial dysfunction,^51^ cardiometabolic diseases,^52^ type 2 diabetes mellitus,^46^ and neurodegeneration.^53^

Stimulated by the finding of potential endoplasmic reticulum–Golgi intermediate compartment/TMED9 involvement and unfolded protein response activation in the kidney biopsies of the 394P *α*-galactosidase A carriers, we investigated several other clinically described *GLA* variants.

First, we studied a female from our cohort with the 143T variant, the most common variant of unknown significance in the United States. She had normal *α*-galactosidase A activity and plasma lyso-globotriaosylceramide levels and presented with late-onset CKD. Her kidney biopsy showed mild interstitial fibrosis and podocytopathy with absence of lysosomal storage. Intracellular *α*-galactosidase A distribution and signs of unfolded protein response activation were very similar to the 394P *α*-galactosidase A carriers. The 143T variant, which we found to be associated with endoplasmic reticulum–Golgi intermediate compartment deposition, has been considered nonpathogenic in several case series studies.^7,43,54^ However, other studies consider the 143T variant to be a cause of non-classic Fabry disease with incomplete age-related and sex-related penetrance and predominantly cardiac manifestations.^55^

We then studied intracellular distribution of several other missense *α*-galactosidase A variants in either stably or transiently transfected HEK 293 cells. Pathogenic variants of *α*-galactosidase A causing classic and non-classic Fabry disease (p.R112H, p.A135V, p.L294X, p.R301Q, and p.R342Q) localized to lysosomes and demonstrated characteristic biochemical abnormalities and histopathologic findings in kidney biopsies. Representative missense *α*-galactosidase A variants associated either with non-classic Fabry disease (p.A97V, p.Q279E, and p.M296I) or variants of unknown significance (p.L394P, p.E66Q, p.R118C, p.A143T, p.R252T, and p.D313Y) localized to the endoplasmic reticulum and endoplasmic reticulum–Golgi intermediate compartment.

Most of the tested missense variants limited *α*-galactosidase A secretion. The intracellular retention of *α*-galactosidase A always activated endoplasmic reticulum stress and unfolded protein response in affected tissues or transfected cells. The intensity of endoplasmic reticulum stress and unfolded protein response varied and was proportional to the secretory deficit of the corresponding variant. There were no differences in endoplasmic reticulum stress and unfolded protein response activation between missense variants retained along the secretory pathway and associated with non-classic Fabry disease (like the 394P) and variants localized to lysosomes and causing the classic Fabry disease (like the 342Q). Thus, AGALopathy seems to be an important component in the pathogenesis of Fabry disease, independent of the enzymatic deficiency and lysosomal storage.

**Figure 8:**
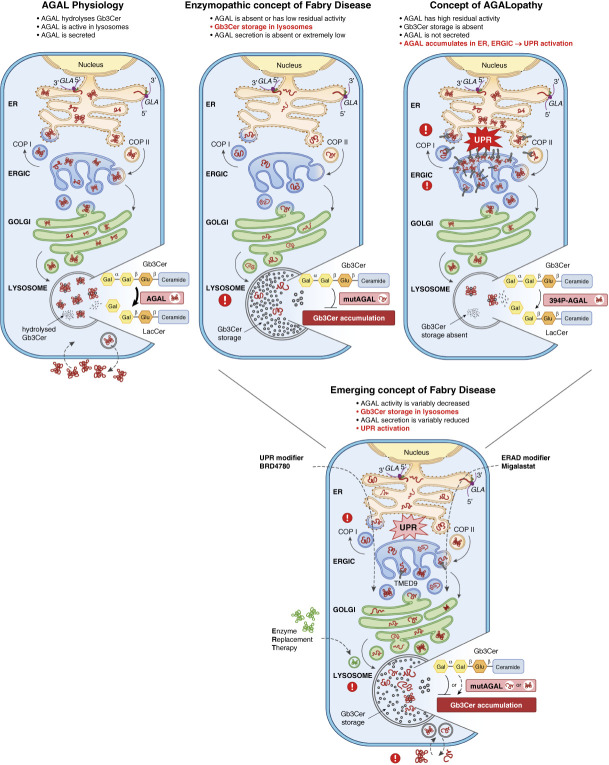
**Endoplasmic reticulum stress and unfolded protein response in Fabry disease.** *α*-galactosidase A is a homodimeric glycoprotein that hydrolyzes the terminal *α*-galactosyl moieties from glycolipids (and some glycoproteins) in lysosomes. During biosynthesis, *α*-galactosidase A is cotranslationally translocated into the endoplasmic reticulum. This process is initiated in the cytoplasm and mediated by the signal peptide (green). In the endoplasmic reticulum, nascent *α*-galactosidase A undergoes a series of post-translational modifications, including removal of the signal peptide, *N*-glycosylation, and chaperone-mediated folding. Properly folded *α*-galactosidase A exits the endoplasmic reticulum in the coat protein complex II (COP II)-coated vesicles and enters the endoplasmic reticulum–Golgi intermediate compartment, where concentration, folding, quality control, and sorting of newly synthesized proteins occurs. Unmaturated proteins are recycled back to the endoplasmic reticulum through coat protein complex I (COP I)-coated vesicles. Mature *α*-galactosidase A traverses through Golgi to lysosomes, where it is activated by the highly acidic pH (between 4.5 and 5.0) and hydrolyses the terminal *α*-galactosyl moieties from glycolipids and glycoproteins. A proportion of *α*-galactosidase A is secreted and re-endocytosed. The classic enzymopathic concept of Fabry disease (middle panel) states that *GLA* variants leading to the loss of *α*-galactosidase A protein or of its enzymatic activity lead to deposition of globotriaosylceramide and other glycosphingolipids in lysosomes. Clinical manifestations are believed to develop as a result of the storage-induced dysfunction of lysosomal and lysosomal-associated cellular compartments and signaling pathways. *α*-galactosidase A secretion is virtually absent. The AGALopathic concept is evidenced by the pathogenic effects of the 394P *α*-galactosidase A variant. Misfolding and abnormal intracellular retention of this missense *α*-galactosidase A variant in endoplasmic reticulum and endoplasmic reticulum–Golgi intermediate compartment disrupt the early secretory pathway and activate the unfolded protein response. At the same time, a portion of the mutated but enzymatically competent *α*-galactosidase A escapes secretory pathway quality control mechanisms and transits to lysosomes, where it prevents accumulation of globotriaosylceramide and related glycosphingolipids. The 394P *α*-galactosidase A is not secreted. Similar effects (retention in endoplasmic reticulum and endoplasmic reticulum–Golgi intermediate compartment, with unfolded protein response activation) can be variably induced also by other missense *GLA* mutations. Secretion of such *α*-galactosidase A variants is decreased proportionally to their defective intracellular trafficking. We believe that the enzymopathic and AGALopathic components should be considered as key contributors to the pathogenesis of Fabry disease. We further propose that both these components contribute to overall pathogenic impacts of individual *α*-galactosidase A variants. Some variants have more enzymopathic and some more AGALopathic nature. Unlike the 394P *α*-galactosidase A variant, which is predominantly AGALopathic, most of the *GLA* missense mutations trigger mixed (enzymopathic and AGALopathic) downstream effects. Whereas Fabry disease patients with predominantly enzymopathic variants are likely to respond well to enzyme replacement therapy or chaperone therapy targeting endoplasmic reticulum–associated degradation, individuals with preferentially AGALopathic variants may be adversely affected by defective trafficking of the misfolded *α*-galactosidase A and augmented consequences of intracellular stress and unfolded protein response. For these variants, compounds like BRD4780, modulating unfolded protein response, may provide new therapeutic opportunities.

We believe that the underlying pathogenesis of Fabry disease symptoms in carriers of missense *α*-galactosidase A variants is related to a combination of decreased enzymatic activity, altered lipid composition of membranes because of limited globotriaosylceramide processing, intracellular *α*-galactosidase A retention, chronic endoplasmic reticulum stress, and unfolded protein response activation. The extent of each factor may be determined by the type of the variant and host factors, with each factor contributing more or less to the clinical findings (Figure 8).

In this investigation, we also found that female patients with the 394P *α*-galactosidase A variant had milder phenotype with later age at onset than male patients. A similar clinical course has also been seen in other female patients with non-classic Fabry disease, often in families with missense variants.^56^ This finding is consistent with the concept of mosaic AGALopathy of the 394P and similar missense *α*-galactosidase A variants rather than loss of enzymatic function of one allele. Moreover, AGALopathy may explain why female patients affected by Fabry disease become symptomatic in the setting of normal or mildly decreased *α*-galactosidase A activity and why the wild-type *α*-galactosidase A secreted from cells expressing the wild-type *GLA* allele does not correct endoplasmic reticulum stress and unfolded protein response within cells expressing the mutated one.

The mechanism of AGALopathy being a lysosomal storage–independent etiologic factor in Fabry disease could also explain why some patients with missense variants treated with recombinant *α*-galactosidase A do not respond to enzyme replacement therapy.^3^ AGALopathy may also explain why some patients treated with Migalastat experienced accelerated loss of kidney function.^57^ This intervention may adversely enhance transport of the misfolded *α*-galactosidase A from endoplasmic reticulum to the endoplasmic reticulum–Golgi intermediate compartment, where it will further augment negative consequences of intracellular stress and unfolded protein response. Moreover, the pathogenic concept of AGALopathy resulting from *α*-galactosidase A misprocessing in the secretory pathway may also apply to phenotypic variability in other lysosomal storage diseases with high residual enzymatic activity(ies).^58^

In conclusion, our results, supported by recent publications,^21,22^ suggest that aberrant intracellular trafficking of some missense *α*-galactosidase A variants and pathogenic interaction within the endoplasmic reticulum–Golgi intermediate compartment/TMED9 leading to maladaptive endoplasmic reticulum stress response may explain some of the varied pathogenic and clinical findings in Fabry disease. Recent research identifying compounds modulating unfolded protein response,^59^ and facilitating removal of unfolded proteins from TMED9 enriched transport vesicles into lysosomes,^39^ provide new opportunities to probe the pathologic and potentially therapeutic implications in Fabry disease.

## Data Availability

The mass spectrometry proteomics data have been deposited to the ProteomeXchange Consortium *via* the PRIDE^60^ partner repository with the dataset identifier PXD033936 and 10.6019/PXD033936. The RNA sequencing data have been deposited to the Gene Expression Omnibus repository with the dataset identifier GSE271756.

## Supplemental Material

This article contains the following supplemental material online at http://links.lww.com/JSN/E952, http://links.lww.com/JSN/E953, http://links.lww.com/JSN/E954, http://links.lww.com/JSN/E955, http://links.lww.com/JSN/E967.

Supplemental Figure 1. LAMP1 immunohistochemistry in kidney biopsies.

Supplemental Figure 2. Structural topology and impact of the L394P variant on *α*-galactosidase A.

Supplemental Figure 3. Immunohistochemical detection of PDIA4/ERp72 in available kidney specimen.

Supplemental Figure 4. Immunohistochemical detection of HSPA5/BiP/GRP78 in available kidney specimen.

Supplemental Figure 5. Immunohistochemical detection of the endoplasmic reticulum stress and activating transcription factor 6 inducible protein CRELD2 in available kidney specimen.

Supplemental Figure 6. Localization of *α*-galactosidase A-FLAG variants transiently expressed in HEK 293 cells.

Supplemental Methods

Supplemental Table 1. List of antibodies.

Supplemental Table 2. Primer and probe sequences for qRT-PCR analysis.

Supplemental Table 3. Clinical presentation and biochemical characteristics of individuals with p.L394P variant.

Supplemental Table 4. Functional annotation of proteomic data.

Supplemental Table 5. Transcriptomic analysis.

Supplemental Table 6. Proteomic analysis.

Supplemental Table 7. *α*-galactosidase A variants of unknown significance and variants causing classic and non-classic Fabry disease characterized in this study.

## References

[1] Cairns T Müntze J Gernert J Spingler L Nordbeck P Wanner C. Hot topics in Fabry disease. Postgrad Med J. 2018;94(1118):709–713. doi:10.1136/postgradmedj-2018-136056 30559317 PMC6581083

[2] Oder D Nordbeck P Wanner C. Long term treatment with enzyme replacement therapy in patients with Fabry disease. Nephron. 2016;134(1):30–36. doi:10.1159/000448968 27576727

[3] Dutra-Clarke M Tapia D Curtin E, . Variable clinical features of patients with Fabry disease and outcome of enzyme replacement therapy. Mol Genet Metab. 2021;132(2):S36. doi:10.1016/j.ymgme.2020.12.069 PMC7788237 33437642

[4] Germain DP Hughes DA Nicholls K, . Treatment of Fabry's disease with the pharmacologic chaperone migalastat. New Engl J Med. 2016;375(6):545–555. doi:10.1056/NEJMoa1510198 27509102

[5] Lenders M Stappers F Brand E. In vitro and in vivo amenability to migalastat in Fabry disease. Mol Ther Methods Clin Dev. 2020;19:24–34. doi:10.1016/j.omtm.2020.08.012 32995357 PMC7490640

[6] Arends M Wanner C Hughes D, . Characterization of classical and nonclassical Fabry disease: a multicenter study. J Am Soc Nephrol. 2017;28(5):1631–1641. doi:10.1681/ASN.2016090964 27979989 PMC5407735

[7] Germain DP Levade T Hachulla E, . Challenging the traditional approach for interpreting genetic variants: lessons from Fabry disease. Clin Genet. 2022;101(4):390–402. doi:10.1111/cge.14102 34927718 PMC9304128

[8] Del Pino M Andrés A Bernabéu AÁ, . Fabry nephropathy: an evidence-based narrative review. Kidney Blood Press Res. 2018;43(2):406–421. doi:10.1159/000488121 29558749

[9] Waldek S Feriozzi S. Fabry nephropathy: a review - how can we optimize the management of Fabry nephropathy? BMC Nephrol. 2014;15:72. doi:10.1186/1471-2369-15-72 24886109 PMC4029839

[10] Germain DP Oliveira JP Bichet DG, . Use of a rare disease registry for establishing phenotypic classification of previously unassigned *GLA* variants: a consensus classification system by a multispecialty Fabry disease genotype-phenotype workgroup. J Med Genet. 2020;57(8):542–551. doi:10.1136/jmedgenet-2019-106467 32161151 PMC7418626

[11] Schiffmann R Fuller M Clarke LA Aerts JMFG. Is it Fabry disease? Genet Med. 2016;18(12):1181–1185. doi:10.1038/gim.2016.55 27195818

[12] Wang RY Lelis A Mirocha J Wilcox WR. Heterozygous Fabry women are not just carriers, but have a significant burden of disease and impaired quality of life. Genet Med. 2007;9(1):34–45. doi:10.1097/gim.0b013e31802d8321 17224688

[13] Vedder AC Linthorst GE van Breemen MJ, . The Dutch Fabry cohort: diversity of clinical manifestations and Gb3 levels. J Inherit Metab Dis. 2007;30(1):68–78. doi:10.1007/s10545-006-0484-8 17206462

[14] Kok K Zwiers KC Boot RG Overkleeft HS Aerts JMFG Artola M. Fabry disease: molecular basis, pathophysiology, diagnostics and potential therapeutic directions. Biomolecules. 2021;11(2):271. doi:10.3390/biom11020271 33673160 PMC7918333

[15] Elsaid HOA Furriol J Blomqvist M, . Reduced α-galactosidase A activity in zebrafish (*Danio rerio)* mirrors distinct features of Fabry nephropathy phenotype. Mol Genet Metab Rep. 2022;31:100851. doi:10.1016/j.ymgmr.2022.100851 35242583 PMC8857658

[16] Braun F Blomberg L Brodesser S, . Enzyme replacement therapy clears Gb3 deposits from a podocyte cell culture model of Fabry disease but fails to restore altered cellular signaling. Cell Physiol Biochem. 2019;52(5):1139–1150. doi:10.33594/000000077 30990584

[17] Eikrem O Skrunes R Tøndel C, . Pathomechanisms of renal Fabry disease. Cell Tissue Res. 2017;369(1):53–62. doi:10.1007/s00441-017-2609-9 28401309

[18] Ledonne NC Fairley JL Sweeley CC. Biosynthesis of alpha-galactosidase-a in cultured chang liver-cells. Arch Biochem Biophys. 1983;224(1):186–195. doi:10.1016/0003-9861(83)90203-5 6307147

[19] Lemansky P Bishop DF Desnick RJ Hasilik A von Figura K. Synthesis and processing of alpha-galactosidase A in human fibroblasts. Evidence for different mutations in Fabry disease. J Biol Chem. 1987;262(5):2062–2065. PMID: 3029062 3029062

[20] Ishii S Chang HH Kawasaki K, . Mutant alpha-galactosidase A enzymes identified in Fabry disease patients with residual enzyme activity: biochemical characterization and restoration of normal intracellular processing by 1-deoxygalactonojirimycin. Biochem J. 2007;406(2):285–295. doi:10.1042/BJ20070479 17555407 PMC1948963

[21] Riillo C Bonapace G Moricca MT Sestito S Salatino A Concolino D. c.376A>G, (p.Ser126Gly) Alpha-Galactosidase A mutation induces ER stress, unfolded protein response and reduced enzyme trafficking to lysosome: possible relevance in the pathogenesis of late-onset forms of Fabry Disease. Mol Genet Metab. 2023;140(3):107700. doi:10.1016/j.ymgme.2023.107700 37774431

[22] Consolato F De Fusco M Schaeffer C, . α-Gal A missense variants associated with Fabry disease can lead to ER stress and induction of the unfolded protein response. Mol Genet Metab Rep. 2022;33:100926. doi:10.1016/j.ymgmr.2022.100926 36345359 PMC9636577

[23] Mayes JS Scheerer JB Sifers RN Donaldson ML. Differential assay for lysosomal alpha-galactosidases in human tissues and its application to Fabry's disease. Clin Chim Acta. 1981;112(2):247–251. doi:10.1016/0009-8981(81)90384-3 6263521

[24] Kuchar L Berna L Poupetova H, . LysoGb3 quantification facilitates phenotypic categorization of Fabry disease patients: insights gained by a novel MS/MS method. Clin Chim Acta. 2024;561:119824. doi:10.1016/j.cca.2024.119824 38906396

[25] Hartmannova H Piherová L Tauchmannová K, . Acadian variant of Fanconi syndrome is caused by mitochondrial respiratory chain complex I deficiency due to a non-coding mutation in complex I assembly factor NDUFAF6. Hum Mol Genet. 2016;25(18):4062–4079. doi:10.1093/hmg/ddw245 27466185

[26] Kuchar L Asfaw B Rybová J Ledvinová J. Tandem mass spectrometry of sphingolipids: applications for diagnosis of sphingolipidoses. Adv Clin Chem. 2016;77:177–219. doi:10.1016/bs.acc.2016.06.004 27717417

[27] Kuchar L Ledvinová J Hrebícek M, . Prosaposin deficiency and saposin B deficiency (activator-deficient metachromatic leukodystrophy): report on two patients detected by analysis of urinary sphingolipids and carrying novel PSAP gene mutations. Am J Med Genet A. 2009;149A(4):613–621. doi:10.1002/ajmg.a.32712 19267410 PMC3437469

[28] Dostalova G Hlubocka Z Lindner J, . Late diagnosis of mucopolysaccharidosis type IVB and successful aortic valve replacement in a 60-year-old female patient. Cardiovasc Pathol. 2018;35:52–56. doi:10.1016/j.carpath.2018.04.001 29800929

[29] Bolar NA Golzio C Živná M, . Heterozygous loss-of-function SEC61A1 mutations cause autosomal-dominant tubulo-interstitial and glomerulocystic kidney disease with anemia. Am J Hum Genet. 2016;99(1):174–187. doi:10.1016/j.ajhg.2016.05.028 27392076 PMC5005467

[30] Didion JP Martin M Collins FS. Atropos: specific, sensitive, and speedy trimming of sequencing reads. PeerJ. 2017;5:e3720. doi:10.7717/peerj.3720 28875074 PMC5581536

[31] Patro R Duggal G Love MI Irizarry RA Kingsford C. Salmon provides fast and bias-aware quantification of transcript expression. Nat Methods. 2017;14(4):417–419. doi:10.1038/nmeth.4197 28263959 PMC5600148

[32] Love MI Huber W Anders S. Moderated estimation of fold change and dispersion for RNA-seq data with DESeq2. Genome Biol. 2014;15(12):550. doi:10.1186/s13059-014-0550-8 25516281 PMC4302049

[33] Cox J Mann M. MaxQuant enables high peptide identification rates, individualized p.p.b.-range mass accuracies and proteome-wide protein quantification. Nat Biotechnol. 2008;26(12):1367–1372. doi:10.1038/nbt.1511 19029910

[34] Tyanova S Temu T Sinitcyn P, . The Perseus computational platform for comprehensive analysis of (prote)omics data. Nat Methods. 2016;13(9):731–740. doi:10.1038/nmeth.3901 27348712

[35] Subramanian A Tamayo P Mootha VK, . Gene set enrichment analysis: a knowledge-based approach for interpreting genome-wide expression profiles. Proc Natl Acad Sci U S A. 2005;102(43):15545–15550. doi:10.1073/pnas.0506580102 16199517 PMC1239896

[36] Richards S Aziz N Bale S, . Standards and guidelines for the interpretation of sequence variants: a joint consensus recommendation of the American College of Medical Genetics and Genomics and the Association for Molecular Pathology. Genet Med. 2015;17(5):405–424. doi:10.1038/gim.2015.30 25741868 PMC4544753

[37] Christensen EI Zhou Q Sørensen SS, . Distribution of alpha-galactosidase A in normal human kidney and renal accumulation and distribution of recombinant alpha-galactosidase A in Fabry mice. J Am Soc Nephrol. 2007;18(3):698–706. doi:10.1681/ASN.2006080822 17287429

[38] Inoue T Maekawa H Inagi R. Organelle crosstalk in the kidney. Kidney Int. 2019;95(6):1318–1325. doi:10.1016/j.kint.2018.11.035 30878214

[39] Dvela-Levitt M Kost-Alimova M Emani M, . Small molecule targets TMED9 and promotes lysosomal degradation to reverse proteinopathy. Cell. 2019;178(3):521–535.e23. doi:10.1016/j.cell.2019.07.002 31348885

[40] Zivna M Kidd K Zaidan M, . An international cohort study of autosomal dominant tubulointerstitial kidney disease due to REN mutations identifies distinct clinical subtypes. Kidney Int. 2020;98(6):1589–1604. doi:10.1016/j.kint.2020.06.041 32750457 PMC7719087

[41] Park SJ Kim Y Chen YM. Endoplasmic reticulum stress and monogenic kidney diseases in precision nephrology. Pediatr Nephrol. 2019;34(9):1493–1500. doi:10.1007/s00467-018-4031-2 30099615 PMC6370526

[42] Oh-hashi K Koga H Ikeda S Shimada K Hirata Y Kiuchi K. CRELD2 is a novel endoplasmic reticulum stress-inducible gene. Biochem Biophysical Res Commun. 2009;387(3):504–510. doi:10.1016/j.bbrc.2009.07.047 19615339

[43] Lenders M Weidemann F Kurschat C, . Alpha-galactosidase A p.A143T, a non-Fabry disease-causing variant. Orphanet J Rare Dis. 2016;11(1):54. doi:10.1186/s13023-016-0441-z 27142856 PMC4855861

[44] Kim SH Choi SJ. Management of hypertension in Fabry disease. Electrolyte Blood Press. 2023;21(1):8–17. doi:10.5049/EBP.2023.21.1.8 37434805 PMC10329903

[45] Sanchez-Nino MD Ceballos MI Carriazo S, . Interaction of Fabry disease and diabetes mellitus: suboptimal recruitment of kidney protective factors. Int J Mol Sci. 2023;24(21):15853. doi:10.3390/ijms242115853 37958836 PMC10650640

[46] Shrestha N De Franco E Arvan P Cnop M. Pathological β-cell endoplasmic reticulum stress in type 2 diabetes: current evidence. Front Endocrinol (Lausanne). 2021;12:650158. doi:10.3389/fendo.2021.650158 33967960 PMC8101261

[47] Leinekugel P Michel S Conzelmann E Sandhoff K. Quantitative correlation between the residual activity of beta-hexosaminidase A and arylsulfatase A and the severity of the resulting lysosomal storage disease. Hum Genet. 1992;88(5):513–523. doi:10.1007/BF00219337 1348043

[48] Braunstein H Papazian M Maor G Lukas J Rolfs A Horowitz M. Misfolding of lysosomal α-galactosidase a in a fly model and its alleviation by the pharmacological chaperone migalastat. Int J Mol Sci. 2020;21(19):7397. doi:10.3390/ijms21197397 33036426 PMC7583893

[49] Lukas J Cimmaruta C Liguori L, . Assessment of gene variant amenability for pharmacological chaperone therapy with 1-deoxygalactonojirimycin in Fabry disease. Int J Mol Sci. 2020;21(3):956. doi:10.3390/ijms21030956 32023956 PMC7037350

[50] Citro V Cammisa M Liguori L, . The large phenotypic spectrum of Fabry disease requires graduated diagnosis and personalized therapy: a meta-analysis can help to differentiate missense mutations. Int J Mol Sci. 2016;17(12):2010. doi:10.3390/ijms17122010 27916943 PMC5187810

[51] Cunard R. Endoplasmic reticulum stress, a driver or an innocent bystander in endothelial dysfunction associated with hypertension? Curr Hypertens Rep. 2017;19(8):64. doi:10.1007/s11906-017-0762-x 28717886

[52] Ajoolabady A Wang S Kroemer G, . ER stress in cardiometabolic diseases: from molecular mechanisms to therapeutics. Endocr Rev. 2021;42(6):839–871. doi:10.1210/endrev/bnab006 33693711

[53] Wodrich APK Scott AW Shukla AK Harris BT Giniger E. The unfolded protein responses in Health, aging, and neurodegeneration: recent advances and future considerations. Front Mol Neurosci. 2022;15:831116. doi:10.3389/fnmol.2022.831116 35283733 PMC8914544

[54] Klein A Klug K Breyer M, . Genetic variants of unknown significance in alpha-galactosidase A: cellular delineation from Fabry disease. J Inherit Metab Dis. 2024;47(4):805–817. doi:10.1002/jimd.12743 38618884

[55] Smid BE Hollak CEM Poorthuis BJHM, . Diagnostic dilemmas in Fabry disease: a case series study on GLA mutations of unknown clinical significance. Clin Genet. 2015;88(2):161–166. doi:10.1111/cge.12449 25040344

[56] Germain DP. Fabry disease. Orphanet J Rare Dis. 2010;5:30. doi:10.1186/1750-1172-5-30 21092187 PMC3009617

[57] Lenders M Nordbeck P Kurschat C, . Treatment of Fabry's disease with migalastat: outcome from a prospective observational multicenter study (FAMOUS). Clin Pharmacol Ther. 2020;108(2):326–337. doi:10.1002/cpt.1832 32198894

[58] Parenti G Medina DL Ballabio A. The rapidly evolving view of lysosomal storage diseases. EMBO Mol Med. 2021;13(2):e12836. doi:10.15252/emmm.202012836 33459519 PMC7863408

[59] Grandjean JMD Wiseman RL. Small molecule strategies to harness the unfolded protein response: where do we go from here? J Biol Chem. 2020;295(46):15692–15711. doi:10.1074/jbc.REV120.010218 32887796 PMC7667976

[60] Perez-Riverol Y Bai J Bandla C, . The PRIDE database resources in 2022: a hub for mass spectrometry-based proteomics evidences. Nucleic Acids Res. 2022;50(D1):D543–D552. doi:10.1093/nar/gkab1038 34723319 PMC8728295

